# HELP-SEEKING IN A FOREIGN COUNTRY: EXPERIENCES OF OLDER LATINO IMMIGRANTS NAVIGATING ASSISTANCE IN AMERICA

**DOI:** 10.1093/geroni/igad104.1759

**Published:** 2023-12-21

**Authors:** Barbara Mendez Campos, Mary Waters, Saul Ramirez, James Hodges, Rocio Calvo

**Affiliations:** Boston College, Boston, Massachusetts, United States; Harvard University, Boston, Massachusetts, United States; Harvard University, Boston, Massachusetts, United States; Boston College, Boston, Massachusetts, United States; Boston College, Boston, Massachusetts, United States

## Abstract

Access and utilization of assistance programs for older Latino immigrants focus on familismo or heavy reliance on family for help-seeking. The purpose of this paper is to document the different experiences of navigating services, including healthcare, for immigrants without family support. We also investigate the role of immigration status and age at migration on immigrants’ strategies for accessing and using social services. We conducted 141 in-depth interviews with immigrants 60 and over from Cuba, Dominican Republic, El Salvador, Mexico, Puerto Rico, and Venezuela. We used a grounded theory approach to code and analyze the data. Three themes shaped the variation we found in the strategies used by older Latino immigrants without family support: formal assistance rooted in state and local policies; age at migration and the resulting patterns of individual agency and resources, and variation by country of origin that captured legal statuses as well as social networks. Our study provides a deeper understanding of a hard-to-reach population’s access to and utilization of social services. It highlights the heterogeneity of Latino immigrants and elucidates differences from culture-based frameworks to understand Latino immigrants’ underutilization of social services later in life. We argue for a shift to perspectives that address how multiple structural dimensions intersect to impact older immigrants’ use of social services.

